# CRISPR/Cas-edited iPSCs and mesenchymal stem cells: a concise review of their potential in thalassemia therapy

**DOI:** 10.3389/fcell.2025.1595897

**Published:** 2025-09-03

**Authors:** Jiaojiao Shu, Xin Xie, Sixi Wang, Zuochen Du, Pei Huang, Yan Chen, Zhixu He

**Affiliations:** ^1^ Department of Pediatrics, Affiliated Hospital of Zunyi Medical University, Zunyi, China; ^2^ Department of Pediatric, Affiliated Hospital of Zunyi Medical University, Guizhou Children’s Hospital, Zunyi, China; ^3^ Department of Pediatrics, Affiliated Hospital of Guizhou Medical University, Guiyang, China

**Keywords:** thalassemia, gene therapy, iPSC, MSC, complication

## Abstract

Thalassemia, a prevalent single-gene inherited disorder, relies on hematopoietic stem cell or bone marrow transplantation as its definitive treatment. However, the scarcity of suitable donors and the severe complications from anemia and iron overload pose significant challenges. An immediate need exists for a therapeutic method that addresses both the illness and its associated complications. Advancements in stem cell technology and gene-editing methods, such as clustered regularly interspaced short palindromic repeats along with its associated protein (CRISPR/Cas), offer encouraging prospects for a therapy that could liberate patients from the need for ongoing blood transfusions and iron chelation treatments. The potential of genetic reprogramming using induced pluripotent stem cells (iPSCs) to address thalassemia is highly promising. Furthermore, mesenchymal stem cells (MSCs), recognized for their capacity to self-renew and differentiate into multiple lineages that include bone, cartilage, adipose tissue, and liver, demonstrate potential in alleviating several complications faced by thalassemia patients, including osteoporosis, cirrhosis, heart conditions, respiratory issues, and immune-related disorders. In this review, we synthesize and summarize relevant studies to assess the therapeutic potential and predict the curative effects of these cellular approaches.

## 1 Introduction

Initially identified along the Mediterranean coast, thalassemia is considered one of the most common autosomal recessive disorders stemming from single-gene inheritance (Zakaria et al., 2022). Annually, approximately 300,000 to 500,000 infants are born with serious types of homozygous thalassemia, and it is estimated that approximately 7% of the worldwide population holds the thalassemia gene (Liu et al., 2024; Tran et al., 2023; Saleemi, 2014). There are regional differences in China; thalassemia patients or carriers mainly live in the south of the Yangtze River, such as Guangxi Province, where the proportion of carriers reaches 20%–25% (Chen et al., 2022). Major thalassemia patients require blood diffusion and iron chelation therapy to survive, which has no cure other than allogeneic hematopoietic stem cell transplantation (HSCT); however, the challenge of identifying a suitably matched donor significantly restricts the accessibility of this therapeutic approach (Zakaria et al., 2022).

Stem cell therapy utilizes the natural abilities of stem cells, such as their inherent capacity for proliferation, differentiation, and self-renewal, to repair damaged cells and promote the healing of organs, providing therapeutic advantages and improving physical development (Mousaei G et al., 2022). Currently, allogeneic HSCT is the primary stem cell treatment for thalassemia and the only recognized potentially curative method for individuals with transfusion-dependent major thalassemia (Farmakis et al., 2022). Furthermore, various research efforts are investigating the combination of gene therapy and mesenchymal stem cells (MSCs) with induced pluripotent stem cells for treating thalassemia (Li et al., 2022a). Clustered regularly interspaced short palindromic repeats along with its associated protein 9 (CRISPR/Cas9) gene-edited induced pluripotent stem cells (iPSCs) from patients can have normal genes and are hopefully capable of differentiating into normal hematopoietic cells and red blood cells (Zakaria et al., 2022). MSCs, which derive from the mesoderm, exhibit significant differentiation capabilities and low levels of immunogenicity (Rudnitsky et al., 2025). MSCs are valuable for therapeutic applications due to their potential to evolve into various adult stem cells, address complications like liver cirrhosis and osteoporosis, facilitate bone healing and liver function improvement in animal studies, and exhibit minimal immunogenicity, reducing the risk of immune rejection (Chen et al., 2023; Huang et al., 2025; Yang et al., 2020). Nevertheless, the relevant research on treating thalassemia with iPSCs and MSCs remains limited. Therefore, in this review, we aim to summarize studies on the application of these stem cells in thalassemia treatment.

## 2 Thalassemia and its complication

### 2.1 Thalassemia pathogenesis

Thalassemia is considered one of the most common genetic disorders inherited in an autosomal recessive manner, distinguished by a defect in the production (resulting from mutations or deletions) of one or several globin peptide chains (α, β, γ, and δ). This alteration creates an imbalance in the hemoglobin structure, which eventually results in the transformation or destruction of red blood cells (Kattamis et al., 2022). Due to decreased normal hemoglobin levels, red blood cells have a shorter lifespan and reduced oxygen transport capacity, and may rupture when passing through the marrow or spleen, leading to hemolytic anemia (Thiagarajan et al., 2021). Thus, the patients exhibit anemic symptoms, for instance, pallor, developmental retardation, and hepatosplenomegaly, due to chronic hypoxia. These symptoms can have a remission through blood transfusion (Musallam et al., 2024).

### 2.2 Clinical manifestation of thalassemia

Thalassemia can result from mutations or deletions in chromosome 16 or chromosome 11, referred to as α-thalassemia and β-thalassemia, respectively; if both chromosomes are mutated, the condition is known as αβ-thalassemia. In clinical practice, γ-thalassemia, δ-thalassemia, and δβ-thalassemia (or εγδβ-thalassemia) also exist; however, they are less prevalent than α-thalassemia and β-thalassemia (Table 1) (Taher et al., 2021).

**TABLE 1:** Thalassemia types and their genetic defects, clinical manifestations, severity, and complications.

Type and genetic deficiency	Clinical manifestation	Severity	Complication	Reference
α-Thalassemia (α-globin gene deletion or deficiency)	a. Silent type: asymptomatic b. Mild type: mild anemia c. Intermediate type (hemoglobin H disease): moderate anemia (may present with jaundice and hepatomegaly) d. Severe type (hydrops fetalis syndrome): severe fetal anemia, generalized edema, and hepatomegaly, often leading to fetal death	Silent and mild types are mild; intermediate type is moderate; severe type is severe	Jaundice, hepatomegaly, hydrops fetalis syndrome, cardiac enlargement, skeletal deformities, delayed growth and development, and iron overload	Musallam et al. (2024), Li et al. (2024), Winger et al. (2025), Farashi and Harteveld (2018), and Tamary et al. (1993)
β-Thalassemia (β-globin gene deletion or deficiency)	a. Mild type: mild anemia; b. Intermediate type: may present with jaundice, hepatomegaly, and delayed growth and development; c. Severe type: severe anemia, pale complexion, hepatomegaly, jaundice, and poor development, with typical facial features, require long-term blood transfusions	Mild type is mild; intermediate and severe types are more severe	Jaundice, hepatomegaly, delayed growth and development, skeletal deformities, heart enlargement, and iron overload	Bajwa and Basit (2025), Makis et al., 2021; Origa (2017), Cao and Galanello (2010), Dordevic et al. (2025), Langer et al. (1993), and Cappellini et al. (2014)
δβ-Thalassemia (δ- and β-globin gene deletion or deficiency)	Mild anemia	Relatively mild	Slight jaundice and hepatomegaly	De Simone et al. (2022)
γ-Thalassemia (γ -globin chain deletion or deficiency)	Usually presents with mild anemia	Relatively mild	Slight jaundice and hepatomegaly	De Simone et al. (2022)
δ-Thalassemia (δ gene mutation)	Mild anemia	Relatively mild	Slight jaundice and hepatomegaly	De Simone et al. (2022)

α-Thalassemia is caused by insufficient production of α-globin peptides, which arises from genes found on chromosome 16. This chromosome typically harbors four α-globin alleles, and the condition’s severity correlates with the number of affected alleles (Harewood and Azevedo, 2024). A single mutation or deletion, known as silent carrier status, is asymptomatic. When two alleles are compromised, the condition is classified as α-thalassemia minor, which is generally mild or without clinical signs. The intermedia form, known as hemoglobin H disease, occurs when three alleles are defective; it can lead to severe symptoms, with some patients requiring blood transfusions (Musallam et al., 2024). Major α-thalassemia, also known as Hb Bart’s hydrops fetalis, often results in miscarriage in the early stages of pregnancy (Tesio and Bauer, 2023). Pregnant women affected by fetal hydrops may experience a condition known as mirror syndrome, which can lead to maternal edema, proteinuria, and hypertension. Additionally, there is an increased risk of dystocia (difficult labor) and postpartum hemorrhage due to the enlarged placenta (Biswas et al., 2023). Unfortunately, because many couples are unaware of their α-thalassemia carrier status and lack access to prenatal screening, most fetuses with α-thalassemia major (ATM) are diagnosed with hydrops or other abnormalities detected through routine prenatal ultrasound examinations (Winger et al., 2025; Tamary et al., 1993). Another kind of thalassemia in hospital is β-thalassemia, and the global morbidity rate of β-thalassemia is approximately 1.5% (Cri et al., 2019). According to available statistics, approximately 4 million infants are born with β-thalassemia (Kattamis et al., 2020), which is mainly distributed in the Mediterranean and Southeast Asia, and a few β-thalassemia infants die before they are diagnosed in some areas with poor medical resources (Taher et al., 2021; Angastiniotis and Lobitz, 2019). β-Thalassemia is difficult to be detected *in utero*, and most patients begin to present clinical symptoms after 6 months of age. Unlike α-thalassemia, β-thalassemia is divided into three types (Zakaria et al., 2022) based on mutation in two alleles on human chromosome 11 (Origa, 2017). Minor thalassemia, known as β-thalassemia carriers, presents with either mild anemia or no clinical symptoms. In contrast, β-thalassemia intermedia involves individuals who are double heterozygotes and exhibit clinical features ranging from mild to severe, potentially resulting in moderate anemia and hepatosplenomegaly (Origa, 2017). Severe β-thalassemia, also known as Cooley’s anemia (Khan and Shaikh, 2023), is associated with the possibility of severe anemia, jaundice, hepatosplenomegaly, growth retardation, and facial skeletal deformity; these patients require regular lifelong blood transfusions, iron chelation therapy, or hematopoietic stem cell transplantation (Needs et al., 2024). Certain patients with severe β-thalassemia may not survive into their twenties due to complications like arrhythmia and heart failure, which can lead to critical deterioration from iron overload within a span of 6 months (Coates, 2014).

### 2.3 Current treatments of thalassemia

Various therapeutic strategies are available for the management of thalassemia, encompassing blood transfusions, iron chelation therapy, splenectomy, bone marrow transplantation, HSCT, gene editing methods, and the induced synthesis of fetal hemoglobin (Wang et al., 2023; Khan and Shaikh, 2024), and medications such as hydroxyurea (Ali et al., 2021), which prolong the life of the patient at the different way. However, some of the treatments pose some challenges that urgently need to be addressed. For example, blood transfusion and iron removal are two symptomatic treatments for major thalassemia (Hodroj et al., 2023); however, frequent or excessive transfusions can lead to iron overload as iron released from the breakdown of red blood cells may deposit in various tissues and organs, resulting in serious complication.

### 2.4 Complications of thalassemia

Although patients with major thalassemia receive the treatments mentioned above, they may still experience numerous complications, including thromboembolic events, organ dysfunction, endocrinopathies, cardiovascular disease, and osteoporosis (Kattamis et al., 2022; Wood, 2023; Gaudio et al., 2019). A cohort study showed that 480 of 709 patients (67.7%) with β-thalassemia have developed at least one complication and 93 β-thalassemia patients died due to heart diseases (57.0%), complications from bone marrow transplantation (10.8%), infections (8.6%), liver diseases (4.3%), cancers (3.2%), thrombus embolism (2.2%), and severe anemia (1.1%) (Forni et al., 2023). These complications are mostly caused by anemia and the occurrence of iron overload resulting from long-term and massive blood transfusions (Yousuf et al., 2022). The accumulation of iron in various organs could result in several types of organ dysfunction, potentially leading to considerable damage and failure of those organs (Wang et al., 2025a). Complications that may arise from this include arrhythmias, dysfunction of the left ventricle (Fu and Yang, 2025), fibrosis or cirrhosis (Langer et al., 1993), osteoporosis (Bhardwaj et al., 2023), kidney disorders (Demosthen et al., 2019), and abnormalities in respiratory function (Bou-Fakhredin et al., 2023). Additionally, endocrine disorders are largely related to iron accumulation in crucial glands such as the pituitary, thyroid, and adrenal glands, significantly impacting the growth and development of patients with thalassemia (Tenuta et al., 2024; Venou et al., 2024; Evangelidis et al., 2023). In addition to other complications, patients may face infections and Graft-versus-Host Disease (GVHD) (Vento et al., 2006; Akbayram et al., 2022), as well as cancer and alloimmunization, which can further threaten the life of the patient.

## 3 The overview of stem cell therapies for thalassemia

**FIGURE 1:**
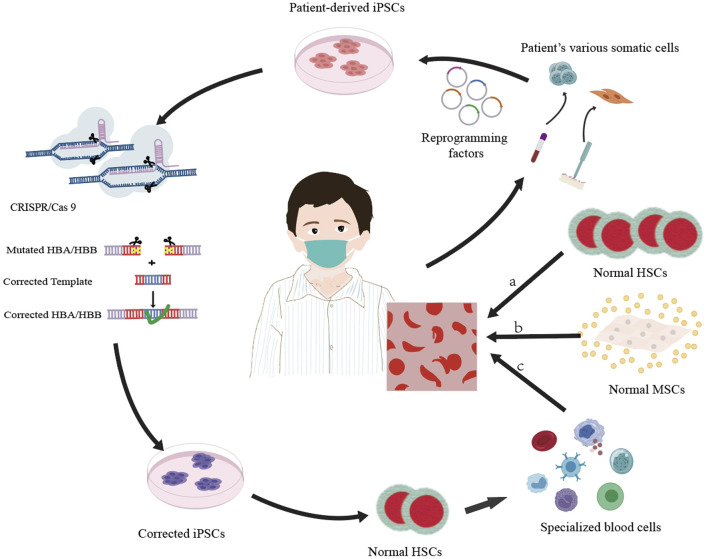
Overview of three cell therapy approaches for thalassemia treatment: a. allogeneic hematopoietic stem cell (HSC) transplantation, where healthy HSCs are transplanted into the patient to replace the defective cells; b. allogeneic mesenchymal stem cell (MSC) transplantation, which utilizes healthy MSCs to enhance the function of HSCs and may help in remedying the condition; c. by using CRISPR/Cas9 technology to modify the mutated HBA/HBB gene in the induced pluripotent stem cells (iPSCs) of the patient, differentiating these corrected iPSCs into healthy hematopoietic stem cells (HSCs), and subsequently transplanting the normal HSCs back into the patient’s body to substitute the defective cells, a targeted gene therapy approach is achieved.

Stem cell treatments encompass allogeneic stem cell therapy, which involves the transplantation of the bone marrow, peripheral blood, and umbilical cord blood sourced from relatives of the patient or from a human leukocyte antigen (HLA)-matched donor (Karponi and Zogas, 2019), as well as autologous stem cell therapy, wherein the patient’s own stem cells are grafted following gene reprogramming (Srivastava and Shaji, 2017); these approaches mainly include hematopoietic stem cell transplantation, mesenchymal stem cell transplantation, and gene-edited stem cell transplantation (Figure 1; Table 2). It is meaningful to develop autologous stem cell transplantation that can correct the genetic defect.

**TABLE 2:** Comparison of the therapies of HSCs, MSCs, and iPSCs.

Stem cell type	Advantage	Disadvantage	Reference
HSCs	a. Currently the only method capable of curing transfusion-dependent thalassemia (TDT) b. Relatively mature technology	a. Donor limitations b. Transplant risks of GVHD. c. Toxicity of conditioning regimens	Wood, (2023), Aljagthmi and Abdel-Aziz (2025), and Hoang et al. (2025)
MSCs	a. Immunomodulation, which can reduce the risk of GVHD. b. Hematopoiesis promotion c. Diverse sources: comprise bone marrow, umbilical cord blood, and adipose tissue, among others	a. Efficacy uncertainty b. Technical challenge c. Long-term safety	Wiewiórska-Krata et al. (2025), Terai et al. (2025), Mousavi et al. (2023), and Levy et al. (2020)
iPSCs	a. Personalized therapy b. Genetic modification: integrating CRISPR/Cas9 with alternative gene editing methods c. Research potential	a. Technical complexity b. Ethical issues: involves ethical debates about embryonic stem cells c. Safety issues: gene editing may introduce off-target effects	Hardouin et al. (2025) and Yamanaka (2020)

### 3.1 The source of stem cells: stem cell bank

Stem cells have the ability to renew themselves and can be classified into two categories depending on their source (Brignier and Gewirtz, 2010), namely, embryonic and adult stem cells. Additionally, stem cells are classified into totipotent, pluripotent, multipotent, and unipotent cells, according to their differentiation potential (Wen and Wang, 2025). A zygote, which is an example of a totipotent stem cell, has the capacity to develop into a fully formed organism. In contrast, a pluripotent stem cell can differentiate into several types of tissues, but it cannot generate an entire individual (Malik and Wang, 2022). Two categories of human pluripotent stem cells exist: human embryonic stem cells (hESCs), originating from the inner cell mass of embryos, and human induced pluripotent stem cells (hiPSCs), obtained through the reprogramming of somatic cells (Tian et al., 2023). In 2006, Takahashi and Yamanaka found that fibroblasts obtained from mice could be converted into specialized cells, reflecting the three germ layers by applying four specific transcription factors: Oct4, Sox-2, Klf4, and c-Myc (Takahashi and Yamanaka, 2006); this technology involving induced pluripotent stem cells has slowly been utilized in the therapy for certain human ailments (Huang et al., 2020). A multipotent stem cell has the ability to develop into one or more specific tissues (Aprile et al., 2024). For instance, hematopoietic stem cells are capable of transforming into erythrocytes, leukocytes, and platelets, whereas mesenchymal stem cells primarily reside in connective tissue and interstitial spaces of organs (Zhang et al., 2018). Unipotent stem cell like myoblast can only differentiate into muscle cells.

Typically, stem cells used to treat thalassemia come from matched donors or the patients themselves, but technological advancements now enable the cultivation of stem cells from biological materials like umbilical cord blood under strict safety and quality regulations (Sharma et al., 2021). Stem cells can be stored in cell banks for future applications (Table 3) and are also used in research and treatment of various diseases (Stacey and Healy, 2021). At present, Zunyi Medical University has cooperated with enterprises to establish the first placental stem cell bank and seven autologous stem cell banks in China, and set up two public stem cell banks in Guizhou Province to provide perinatal stem cell storage services and store important stem cell medicine treatment needs. Ossium Health has established a cryopreserved bone marrow cell repository recovered from deceased organ vertebrae, referred to as hematopoietic progenitor cell bone marrow (Johnstone et al., 2021).

**TABLE 3:** Founding time, source, and reason for establishment of Stem Cell Banks.

Stem cell bank	Founding time	Source	Reason for establishment	Reference
UK Stem Cell Bank(UKSCB)	2003	Approximately 100 research-grade hESC lines and several human pluripotent stem cell lines	Foster the advancement of scientific research and the clinical application of stem cell treatments	O’Shea and Abranches (2020)
Korea National Stem Cell Bank (KSCB)	2005	17 tissue-derived adult stem cells and 228 primary genetic disease cells	Supply stem cell resources, regenerative medicine information, and hESC registry	Kim et al. (2021)
Karolinska Institute Human Embryonic Stem Cell Bank (KISCB)	2002	60 hESC lines	Set up hESC lines for clinical use: remove xeno, chemical conditions, and ensure GMP.	Main et al. (2020)
Spanish National Stem Cell Bank (BNLC)	2006	40 hESC lines and 171 hiPSC lines, including cord blood and adipose-derived MSCs	Advance stem cell and regenerative medicine; establish hiPSC bank from homozygous cord blood	Aran et al. (2020)

### 3.2 Allogeneic stem cell transplantation for thalassemia

Currently, the only curative treatment for severe thalassemia is allogeneic hematopoietic stem cell transplantation (Srivastava and Shaji, 2017). This process involves utilizing hematopoietic stem cells obtained from external sources, such as the bone marrow, peripheral blood, or umbilical cord blood of relatives (Wei et al., 2024). The aim is to restore the blood and immune systems of the patient, with the success of the treatment being affected by variables including the recipient’s age, compatibility with the donor, and medical care provided prior to the transplant.

#### 3.2.1 Bone marrow transplantation

Clinically, the initiation of bone marrow transplantation for disease treatment occurred in 1957 (Simpson and Dazzi, 2019). Bone marrow serves as a primary source of hematopoietic stem cells (Agarwal et al., 2023), and this procedure stands as the definitive treatment for thalassemia, particularly among individuals suffering from severe β-thalassemia (Ali et al., 2021). Bone marrow transplantation can help patients with thalassemia restore normal red blood cell production by providing healthy hematopoietic stem cells, thereby improving anemia symptoms and potentially achieving a cure, but it also involves inherent challenges and risks (Oikonomopoulou and Goussetis, 2021). Post-transplantation, patients might experience complications, including GVHD and hepatobiliary disorders (Dezan et al., 2023).

#### 3.2.2 Umbilical cord blood transplantation

Umbilical cord blood contains a large number of hematopoietic stem cells, and it can be used in patients with no compatible donor (Hordyjewska et al., 2015). For more than 30 years, the transplantation of umbilical cord blood has been used in medical practices (Gupta and Wagner, 2020). Although it was initially regarded as medical waste, a thorough investigation into its biological characteristics and immunogenicity has revealed that umbilical cord blood is abundant in hematopoietic stem cells, which tend to proliferate and survive more effectively than other types of stem cells (Zhu et al., 2021a). Furthermore, it exhibits low immunogenicity, does not require strict HLA matching, causes no harm to donors, and is associated with a lower incidence of GVHD in transplant recipients (Zhu et al., 2021b). Compared to bone marrow, the matching process for cord blood demands a lesser degree of site matching, thereby making it easier to find a compatible donor (Sanchez-Petitto et al., 2023). Although umbilical cord blood transplantation has distinct benefits, it is utilized less often in clinical settings than bone marrow and peripheral blood hematopoietic stem cell transplants. This is attributable to factors such as the possibility of delayed engraftment, the risk of graft failure, elevated non-relapse mortality rates, a heightened susceptibility to infections, and the considerable expenses involved in procuring umbilical cord blood (Sirinoglu Demiriz et al., 2012; Kindwall-Keller and Ballen, 2020).

#### 3.2.3 Peripheral blood stem cell transplantation

The use of peripheral blood stem cell transplantation (PBSCT) from a matched sibling donor has been suggested as an alternative to bone marrow transplantation to reduce the risk of transplant failure in patients with major thalassemia (Körbling et al., 2011). PBSCT is a vital treatment method for thalassemia, which involves collecting hematopoietic stem cells from a donor, who can be the patient themselves or a matched donor, and then transfusing them intravenously into the patient’s body (Angelucci et al., 2014). These stem cells migrate to the bone marrow, replacing the patient’s abnormal hematopoietic cells and restoring normal red blood cell production (Körblin et al., 2011). This method holds promise for treating thalassemia; however, it requires finding an appropriate donor and addressing complications associated with transplantation (Angelucci et al., 2014). A successful transplant can greatly enhance the patient’s quality of life, potentially decreasing or even removing the requirement for blood transfusions and iron chelation therapy. However, not all of the transplant patients can be saved; some of them die from complications after transplantation like GVHD, osteonecrosis, and infection (Kuci et al., 2020). However, the failure rate of allograft is approximately 25% (Marziali et al., 2017). Transplants of hematopoietic stem cells sourced from siblings who share identical HLA profiles show notably improved success rates when contrasted with those obtained from unrelated donors. However, it is important to note that merely 35%-40% of patients with thalassemia are able to find a sibling donor with matching HLA (O’Reilly, 1983). In addition, Jagasia demonstrated that approximately 40% HSCT of identical siblings will get GVHD, whereas 59% will get GVHD in the transplantation of the unrelated donor hematopoietic stem cells (Jagasia et al., 2012). A research indicates that approximately 15%-16% of patients experience grades II-IV acute GVHD and 4%-12% experience chronic GVHD after transplantation for thalassemia (Mahmoud et al., 2015).

### 3.3 Autologous stem cell transplantation and gene-editing therapy for thalassemia

Transplantation of autologous hematopoietic stem cells or therapy involving gene editing represents a novel approach that functions by modifying a patient’s own blood stem cells to correct the genetic mutation responsible for thalassemia (Zeng et al., 2023). This approach avoids donor-matching problems in allogeneic transplantation and may reduce the occurrence rate of GVHD and infectious diseases (Li and Yang, 2023). Autologous hematopoietic stem cell transplantation typically involves collecting hematopoietic stem cells from a person, using gene-editing techniques such as CRISPR-Cas9 to amend the genetic mutation, and then reinfusing the altered stem cells into the same individual (Wilkinson et al., 2021).

## 4 CRISPR/Cas-edited induced pluripotent stem cells

So far, gene-editing technology to treat thalassemia has been widely used in research and is considered one of the most promising therapies for diseases resulting from single-gene inheritance (Khiabani et al., 2023). Gene editing represents an advanced therapeutic approach that focuses on cultivating patients’ hematopoietic stem cells. Following this, the edited stem cells containing the corrected gene are injected back into the patients’ bodies to achieve complete healing (Ali et al., 2021). At present, gene therapy is in the experimental stage and has made some progress. Some research works have pointed out that gene-editing technologies like CRISPR/Cas 9 (Zeng et al., 2023) can accurately modify or replace defective genes and ultimately restore the function of red blood cells (Traeger-Synodinos et al., 2024). Although gene therapy offers certain advantages over other therapies, it is not suitable for all thalassemia patients, and its high cost makes it inaccessible to many patients; moreover, the therapy becomes ineffective if the corrected gene is inserted off-target (Christakopoulos et al., 2023).

### 4.1 CRISPR/Cas9 gene-editing technology

The CRISPR/Cas9 system was initially used by bacteria and archaea for adaptive immune responses against foreign DNA sources like plasmids and viruses. It has now become a potent instrument for gene editing (Deltcheva et al., 2011). With the introduction of CRISPR/Cas9 technology in 2012 (Jinek et al., 2012), this method has shown considerable promise in addressing genetic disorders, including thalassemia. Although CRISPR/Cas9 has limitations in off-target effects and editing efficiency, its flexibility and programmability have spurred next-generation, more precise tools like Base Editing, offering broader strategies for treating monogenic diseases such as thalassemia (Table 4) (Greco et al., 2024). The CRISPR/Cas9 system, guided by RNA, is notable for being easy to use, economical, and versatile (Anurogo et al., 2021). However, despite its broad application prospects, gene therapy raises concerns about its safety and ethical implications, particularly the risk of off-target effects, which require rigorous ethical review and regulatory oversight (Hryhorowicz et al., 2023).

**TABLE 4:** Comparison of gene therapy strategies for thalassemia.

Therapeutic approach	Principle	Advantage	Disadvantage	Correction of known pathogenic mutations	Safety	Reference
CRISPR/Cas9 gene editing	Utilizes the CRISPR/Cas system to directly repair or insert target genes, correcting mutations	a. Precise correction of mutations b. Low risk of immune reactions c. Applicable to various cell types	a. Unintended effects b. The efficiency of delivery and specificity for cells need additional refinement	Suitable for cases requiring long-term stable expression of normal genes	a. Off-target effects and long-term safety b. Delivery system safety requires optimization	Paolini Sguazzi et al. (2021), Wang et al. (2025b), Butterfield et al. (2025), Gao et al. (2025), John and Czechowicz (2025), and Ali et al. (2025)
Lentiviral vectors	Introduces normal genes into cells via viral vectors to replace defective genes	a. Efficient gene insertion b. Long-term stable expression c. Extensive clinical experience	a. Potential for immune reactions b. Risk of insertional mutagenesis	Correction of single-base mutations	a. Long-term monitoring required b. Safety relatively mature but still with risks	Williams et al. (2025), Ottaviano and Qasim (2025), and Hackett and Crystal (2025)
Base editing	Chemically alters one base to another, which can be divided into two primary categories: those that target DNA and those that focus on RNA.	a. No double-strand breaks b. High efficiency for single-base conversions	a. Lower technological maturity b. Limited scope of application	a. Still in preclinical research stages b. Not yet widely applied clinically	a. Long-term stability and potential side effects b. Off-target effects	Porto et al. (2020), Komor et al. (2016), Gaudelli et al. (2017), and Rees and Liu (2018)

### 4.2 Human induced pluripotent stem cell

In 2006, Shinya Yamanaka et al. found that it was possible to reprogram adult skin fibroblasts into hiPSCs by incorporating four transcription factors: Oct4, Sox2, Klf4, and c-Myc (Takahashi and Yamanaka, 2006). iPSCs closely resemble embryonic stem cells regarding their morphological characteristics, patterns of gene expression, and capabilities for differentiation, which have been validated in various 3D cardiac tissue models (Campostrini et al., 2021). These cells exhibit remarkable self-renewal capabilities and have the potential for multipotent differentiation, rendering them a valuable resource for various applications, including regenerative medicine, drug development, and disease modeling (Poetsch et al., 2022). iPSCs derived from an individual’s own cells provide considerable medical benefits by reducing the likelihood of immune rejection. Their capacity for self-renewal and differentiation into diverse cell types positions them as essential tools in regenerative medicine, drug discovery, and disease modeling (Cerneckis et al., 2024). iPSCs possess the ability to replicate and grow indefinitely in a laboratory setting, thus offering a substantial source of cells for applications in tissue engineering and cellular therapies (Mattis and Svendsen, 2011). This property is essential for producing the required cell populations for regenerative medicine (Rowe and Daley, 2019). However, iPSCs encounter challenges including potential genetic mutations and chromosomal abnormalities arising from reprogramming, which can compromise cell quality and safety (Kavyasudha et al., 2018). Additionally, effective differentiation into specific cell types and achieving targeted differentiation with functional integration *in vivo* present ongoing research challenges (Lee and Son, 2021).

#### 4.2.1 Gene-editing strategies

Technologies for gene editing offer considerable promise for utilizing iPSCs (McTague et al., 2021). For instance, CRISPR/Cas9 tools can be utilized to modify iPSCs by either knocking out genes that hinder nerve regeneration (like Nogo-A) or inserting genes that encourage nerve repair (such as the neurotrophic factor BDNF) (Hsu et al., 2019). Additionally, for allogeneic iPSCs, HLA genes can be edited to match those of the patient or to create universal donor cells, thereby reducing the risk of immune rejection (Xu et al., 2019; Song et al., 2022). It is necessary to conduct sequencing, flow cytometry and other analyses on the verified edited iPS cells to verify the accuracy of the modification and the normal differentiation ability of the cells (Maurissen and Woltjen, 2020).

#### 4.2.2 Strategy for differentiating hiPSCs into HSCs

A fundamental approach to differentiating iPSCs into hematopoietic stem cells (HSCs) involves the creation of embryoid bodies (EBs), induction of mesoderm, formation of hematopoietic endothelium, and the process of endothelial-to-hematopoietic transition (EHT) (Ng et al., 2024). This process also encompasses the utilization of various elements, such as cytokines [interleukin (IL)-3, IL-6], dexamethasone, stem cell factor (SCF), recombinant human erythropoietin (EPO), vascular endothelial growth factor (VEGF), insulin-like growth factor I (IGF-I), Fms-like tyrosine kinase 3 (FLT3), bone morphogenetic protein 4 (BMP4), albumin, and transferrin (Lapillonne et al., 2010; Bernecker et al., 2019; Rowe et al., 2016; Oguro, 2019). In addition, retinoic acid and its precursors (such as retinol) play important roles in the mesoderm patterning stage, significantly enhancing the efficiency of iPSC differentiation into hematopoietic cells (Grace et al., 2018). However, the differentiation of iPSCs into functional HSCs still faces challenges such as cell functional stability and immunogenicity (Wattanapanitch et al., 2019).

### 4.3 CRISPR/Cas-edited induced pluripotent stem cell for thalassemia

iPSCs utilizing CRISPR/Cas gene-editing techniques present an innovative strategy for treating thalassemia (Wattanapanitch et al., 2018). In the context of thalassemia management, the CRISPR/Cas technology is capable of correcting genetic mutations within iPSCs, which restores their erythropoietic function and allows for the production of healthy red blood cells through *in vitro* cultivation and differentiation into erythroid lineages (Xie et al., 2014). These differentiated red blood cells can subsequently be reintroduced into the patient, acting as a form of cellular therapy that either supplements or replaces the malfunctioning cells, thereby alleviating the symptoms related to the genetic mutation (Ou et al., 2016). This approach is particularly advantageous as it utilizes the patient’s own cells, reducing the risk of immune rejection. Moreover, this technique could be applied to the treatment of various other genetic conditions (Li et al., 2023). The CRISPR/Cas9 technique for editing genes related to thalassemia poses numerous challenges, especially regarding safety considerations and the potential long-term consequences of this new technology, which have not yet been completely determined (Frangoul et al., 2021). Overall, using CRISPR/Cas gene-editing techniques on induced pluripotent stem cells exhibits potential as a therapy for thalassemia; nonetheless, further studies and confirmations are necessary prior to its widespread adoption in clinical applications (Zeng et al., 2023).

#### 4.3.1 Delivery methods

The methods of delivering the CRISPR/Cas system play a vital role in the effectiveness of gene editing (Du et al., 2023). Typical methods of delivery encompass viral vectors such as adeno-associated virus (AAV) (Naso et al., 2017), adenovirus (AdV), lentivirus, Sendai virus, and retrovirus (Tong et al., 2019; Park et al., 2016), along with nonviral systems including lipid nanoparticles (Li et al., 2018; Sinclair et al., 2023). Although viral vectors offer high delivery efficiency, they are associated with risks of immune responses and insertional mutagenesis. In contrast, nonviral delivery systems are safer but less efficient (Seijas et al., 2025).

#### 4.3.2 Editing efficiency

Editing efficiency directly impacts the therapeutic outcome. Improvements in the efficiency of the CRISPR/Cas9 system can be achieved through the optimization of sgRNA design, expression levels of Cas9 protein, and the selection of cell types (Agrawal et al., 2021). Utilizing high-precision variants of Cas9, such as eSpCas9 or SpCas9-HF1, can reduce off-target effects while also enhancing the safety of the editing procedure (Kleinstiver et al., 2021; Yin et al., 2016). In iPSCs, increased editing efficiency ensures more successful cell repair, fewer residual mutant cells, and better therapeutic efficacy, as shown by Singh et al. (2024), using p53 inhibition and pro-survival molecules to achieve over 90% CRISPR/Cas9 efficiency.

#### 4.3.3 Differentiation into functional hematopoietic stem cells

The process of transforming corrected iPSCs into functional hematopoietic stem cells represents a crucial aspect of cellular therapy (Table 5). At present, by replicating the *in vivo* microenvironment that supports hematopoietic development and integrating specific cytokines (like BMP4, SCF, and TPO) with small molecular compounds (Bello et al., 2024; Jeong et al., 2020a; Kayama et al., 2024), iPSCs can be effectively guided to develop into the hematopoietic lineage. Further optimization of differentiation protocols, such as adjusting culture conditions, adding functional small molecules, and utilizing three-dimensional culture systems, holds promise for improving differentiation efficiency and cell quality (Rahman et al., 2025; Meng et al., 2014). Additionally, verifying the functionality of the differentiated cells through flow cytometry and *in vivo* transplantation experiments is an essential step to ensure therapeutic efficacy (Bhattacharya et al., 2014).

**TABLE 5:** The treatment and therapeutic effect of using iPSC to treat the thalassemia patients.

Disease	iPSC source	Treatment	Therapeutic effect	Reference
β-Thalassemia (homozygous 41/42 deletion)	β-Thal patient	CRISPR/Cas9+ iPSCs	Effectively fixes β-thal mutations in patient iPSCs	Ou et al. (2016)
β-Thalassemia [β17/17 (A→T) in HBB]	β-Thal patient	CRISPR/Cas9	Edited cells show normal karyotypes, pluripotency, and no off-target.	Song et al. (2015)
β-Thalassemia [IVS2-654(C>T)]	β-Thal patient	CRISPR/Cas9 + piggyBac	CRISPR/Cas9 detected off-target.	Xu et al. (2015)
HbE mutation	β-Thal patient	CRISPR/Cas9 plasmid + ssODN	Achieves one-step HbE correction in iPSCs	Wattanapanitch (2021)
β-Thalassemia (a homozygous β41-42 del and heterozygous Westmead mut in HBA2)	Fetal amnion	CRISPR/Cas9+ iPSCs	Mutations fixed; hiPSCs kept normal pluripotency and could become hematopoietic progenitors	Li et al. (2022a)
β-Thalassemia [β17/17 (A→T) in HBB]	β-Thal patient	CRISPR/Cas9 + iPSCs	Normal karyotype, maintained pluripotency, and no off-target effects	Song et al. (2015)
β-Thalassemia [−28 (A>G) and the 4-bp (TCTT) del at CD41-42 in exon 2]	β-Thal patient	CRISPR/Cas9 + iPSCs + piggyBac	Seamless HBB mutation correction via HDR; no off-target effects	Xie et al. (2014)
β-Thalassemia [4-bp del (–TCTT) and (–CTTT) at CD41-42 mut]	β-Thal patients	CRISPR/Cas9 + ssODNs	Repaired cells had normal β-globin transcripts, low mutation load, and no off-target mutagenesis	Niu et al. (2016)
β-Thalassemia (CD26 G>A mut in in HBB)	HbE/β-thalassemia patient’s dermal fibroblasts with CD41/42 and CD26 mut	Cas9 + ssODNs via HDR	HBB protein restored; single CD26 allele fix normalizes β-globin in HbE/β-thalassemia	Wattanapanitch et al. (2018)

### 4.4 Barriers to translating research into clinical applications

#### 4.4.1 Technical challenges and strategies for mitigation

One of the primary challenges in applying gene-editing technologies such as CRISPR/Cas9 to clinical treatments is the technical barriers associated with off-target effects (Concordet and Haeussler, 2018). Unplanned alterations to genes that are not the intended targets may heighten the risks linked to therapeutic treatments. To reduce these unintended effects, various strategies have been formulated.

High-fidelity Cas9 variants: the use of high-fidelity Cas9 variants (Zuo et al., 2022), such as eSpCas9, SpCas9-HF1, dCas9-FokI and evoCas9 (Allemailem et al., 2023; Wyvekens et al., 2015), has been extensively acknowledged in academic writings as an approach to enhance the accuracy of CRISPR/Cas9-editing systems (Naeem et al., 2020). The highly accurate version of Cas9, referred to as Hypa-Cas9, has demonstrated enhanced on-target effectiveness in human cells while minimizing off-target impacts (Chen et al., 2017).

Optimized sgRNA design: the careful design of single-guide RNA (sgRNA) improves the accuracy of gene editing and reduces off-target consequences (Doench et al., 2016). Using bioinformatic tools to predict and select sgRNA sequences with low off-target risks is an effective method to improve specificity (Concordet and Haeussler, 2018; Doench et al., 2016). Recent developments in the methods of delivering CRISPR/Cas9 for therapeutic gene editing in stem cells have been examined, emphasizing the significance of well-designed sgRNA in improving the efficiency of genome editing while minimizing off-target impacts (Lotfi et al., 2023).

Delivery approaches for CRISPR/Cas9 systems: enhancing the delivery mechanisms for the CRISPR/Cas9 system, using nonviral strategies like lipid nanoparticles (Kazemian et al., 2022) or electroporation (Hussen et al., 2024) as nonviral delivery systems, can lead to improved editing efficiency and a decrease in off-target effects.

Precise control of editing windows: introducing mutations into the deaminase allows for the narrowing of the editing window while still preserving significant editing activity (Jeong et al., 2020b). A study used adenine base editors (ABEs) to accurately correct the IVSI-110(G>A) mutation associated with beta-thalassemia, attaining a 90% efficiency in editing (Naiisseh et al., 2024). By precisely controlling the editing window, that is, performing edits during specific cell cycle stages, off-target effects can be minimized, as certain cell cycle stages are more precise in DNA damage repair mechanisms (Fichter et al., 2023).

Using these approaches allows us to more precisely modify mutations that lead to diseases, like thalassemia, while minimizing the potential for off-target effects.

#### 4.4.2 Ethical and regulatory considerations

The translation of gene-editing technologies into clinical applications faces significant technical, ethical, and regulatory challenges (Ledford, 2020). The swift advancement of these technologies raises ethical concerns regarding safety, equity, privacy, and societal impact (Greely, 2019). The establishment of a unified global regulatory system faces challenges, as more countries become open to gene-editing technology (Sprink et al., 2022). Ethical reviews are crucial for ensuring research integrity and participant rights, and research workers must comply with laws including clinical trial regulations and data protection (Joseph et al., 2022). Public engagement and transparency are key factors to fostering understanding and discourse on these issues (Parikh et al., 2025). Overcoming these challenges is vital for advancing gene-editing technology in clinical settings (Wiley et al., 2025), potentially offering novel treatments for diseases like thalassemia.

## 5 Biological characteristics and function of MSCs

### 5.1 Biological properties

MSCs are a type of multipotent stem cell that was first discovered in the bone marrow by Friedenstein and are derived from the dental pulp, umbilical cord blood, amniotic membrane, placenta, mobilised peripheral blood, synovium and synovial fluid, endometrium, skin and muscle (Berebichez-Fridman and Montero-Olvera, 2018), which possess the ability to self-renew and differentiate into various cell types (Kolf et al., 2007). MSCs, mainly found in connective tissue and apparatus mesenchyme, are an important cell repository involved in tissue regeneration; MSCs are also a significant type of seed cell used in tissue engineering (Adil et al., 2022). MSCs proliferate rapidly and are easily isolated and cultured. These cells may be sourced from multiple origins, including bone marrow, periosteum, adipose tissue (Deo et al., 2022), umbilical cord tissue (Wu et al., 2023), dental tissue (Gan et al., 2020), and amniotic fluid (Gholami Farashah et al., 2023). Additionally, MSCs contribute to immune modulation by interacting with T cells (Kuca-Warnawin et al., 2021), B cells, NK cells (Abbasi et al., 2022), and other types of immune cells. Importantly, MSCs have little or no immunogenicity (Chen et al., 2024b) and the tolerance of immunity, which can reduce the risk of graft rejective reaction. Recently, a study found that higher-level TNF-α-induced protein 6 (TSG6) enhanced the anti-inflammatory function of CD317(+) MSCs, suggesting that CD317(+) MSCs may be a promising candidate for treating the immune-related diseases (Song et al., 2024). MSCs can repair tissues and differentiate into osteoblast, chondroblast, nerve cells, myoblast, and adipose tissues (Kim et al., 2023). Given the capabilities of MSCs, extensive research has been conducted on them, leading to their increasing implementation in clinical settings.

### 5.2 The possibility of MSC transplantation to treat thalassemia

A study has indicated that MSCs can enhance the homing of hematopoietic stem cells and boost their hematopoietic capacity (Aqmasheh et al., 2017). Given their role in immunological regulation, MSCs can inhibit NK cell activity and reduce T-cell proliferation by releasing specific cytokines and facilitating cell interactions, which is beneficial for minimizing rejection responses and enhancing survival rates (Johnstone et al., 2021). In patients with β-thalassemia, the MSCs in the bone marrow are functionally impaired due to iron overload and oxidative stress, leading to a decrease in their proliferation, differentiation capacity, and hematopoietic supportive function (Cri et al., 2019). The alteration of the bone marrow microenvironment in patients with thalassemia leads to a significant increase in the lipid, protein, glycogen and nucleic acid content of bone marrow mesenchymal stem cells (BM-MSCs), which is related to enhanced cell proliferation and bone marrow activity (Aksoy et al., 2012). Moreover, studies have shown that the vertebral body - adherent mesenchymal stromal cells (vBA - MSCs) extracted from donor vertebral fragments have similar characteristics to traditional bone marrow - derived mesenchymal stromal cells (BM - MSCs), but with a significantly higher abundance (Johnstone et al., 2020). Additionally, they are matched with hematopoietic progenitor cells (HPCs), which helps promote the formation of mixed chimerism, enhance peripheral immune regulatory functions, and improve the safety of transplantation (Johnstone et al., 2021).

### 5.3 Research on using MSCs to treat the complication of thalassemia

**FIGURE 2:**
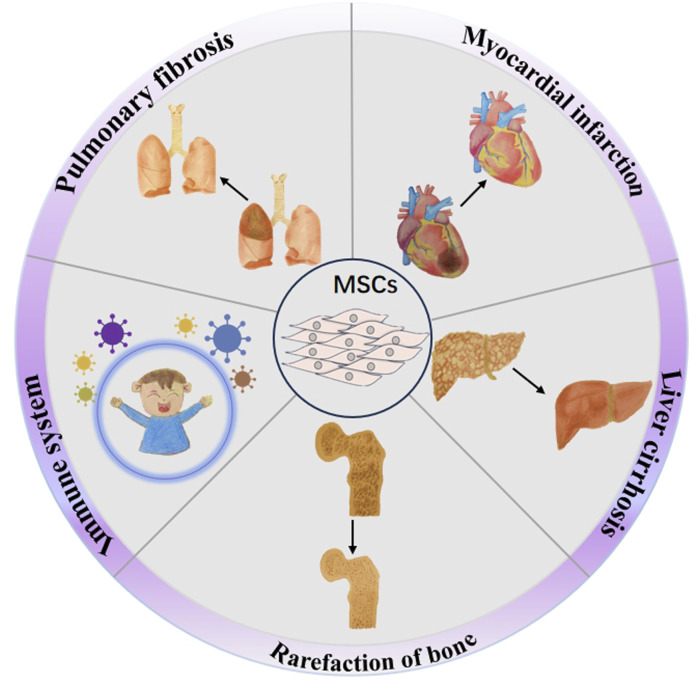
Therapeutic potential of MSCs in addressing thalassemia-related complications. The potential of MSCs to treat the complications of thalassemia including myocardial infarction, liver cirrhosis, rarefaction of bone, pulmonary fibrosis, and immune system damage.

MSCs have shown potential therapeutic values in treating multiple complications of thalassemia, including heart disease (Szaraz et al., 2017), liver disease (Banas et al., 2007; Ortuño-Costela et al., 2025), bone destruction (Afflerbach et al., 2020), lung disease (Mansouri et al., 2019), endocrine abnormalities (Sarvari et al., 2021), and other diseases (Hoang et al., 2022) (Figure 2). First, thalassemia patients face a long-term anemic state and need regular blood transfusion, which may lead to increased heart burden, causing heart enlargement, cardiac hypertrophy, and other heart diseases (Berdoukas et al., 2015), and MSCs play a role in protecting and repairing the heart. They facilitate the regeneration and repair of injured myocardial tissue, enhancing cardiac function by differentiating into both cardiomyocytes and vascular smooth muscle cells (Ala, 2023; An et al., 2025). Second, chronic anemia and increased red blood cell destruction in individuals with thalassemia can place a greater strain on the liver, potentially leading to liver diseases such as hepatic fibrosis (Papastamataki et al., 2010). MSCs have the ability to release various growth factors and cytokines, aiding in the regeneration and repair of liver cells, minimizing liver inflammation, and enhancing liver function (Rostami et al., 2021; Wen et al., 2025). Third, patients with thalassemia may have bone destruction and osteoporosis due to chronic anemia and myelodysplasia (Bhardwaj et al., 2023), and MSCs have the ability of osteogenic differentiation, can promote the regeneration and repair of bone tissue, can increase bone density and bone strength, and can improve bone condition (Jiang et al., 2021). Fourth, thalassemia patients receiving chronic transfusions are at risk of iron overload and pulmonary fibrosis (Carnelli et al., 2003). MSCs have the potential to address these respiratory concerns by transforming into alveolar and lung epithelial cells, facilitating the repair of lung tissue, minimizing fibrosis, and enhancing overall lung performance (Doherty et al., 2024; Antoniou et al., 2010), offering a new therapeutic strategy for lung diseases associated with thalassemia. Additionally, thalassemia patients may experience endocrine system dysfunction due to long-term anemia, malnutrition, and other factors (Carsote et al., 2022). Endocrine abnormalities and MSCs can regulate the balance between the immune and endocrine systems, promote the functional recovery of endocrine glands, and improve the symptoms associated with endocrine abnormalities (Hoang et al., 2022). In addition to these challenges, individuals with thalassemia who have undergone blood transfusions, iron chelation therapy, or bone marrow transplantation might experience adverse effects resulting from these treatments (Yousuf et al., 2022). Current research on using MSCs to treat thalassemia-related complications is still in the early stages, with further exploration needed in areas such as specific treatment mechanisms, optimal administration methods, and efficacy evaluation (Li et al., 2024). Moreover, addressing the challenges related to the sourcing, preparation, and quality assurance of MSCs is essential to ensure the safety and effectiveness of these treatments (Guadix et al., 2019). In summary, MSCs demonstrate considerable potential as a therapy for various complications linked to thalassemia, and it is expected that they will provide enhanced treatment alternatives for these individuals moving forward.

### 5.4 Challenges in translational application: technical, ethical, and regulatory aspects

During the process of transplanting allogeneic MSCs into patients, challenges in technology, ethics, and regulations must be addressed (Velikova et al., 2024). Technically, it is crucial to guarantee the viability, homing ability, and long-term stability of the cells, which includes optimizing cell isolation, expansion, and cryopreservation techniques (Wang and Li, 2024). Additionally, utilizing the patient’s own MSCs after genetic modification and subsequent autologous transplantation offers a promising approach to potentially reduce the risk of immune rejection and GVHD (Večerić-Haler et al., 2022; Li et al., 2022b). Ethical considerations involve obtaining fully informed consent from patients, safeguarding patient privacy, and ensuring equitable access to treatment (Lo et al., 2009). Regulatory compliance requires adherence to international and local regulatory standards, navigating the approval process, and conducting long-term surveillance to evaluate the safety and efficacy of the therapy (Farmakis et al., 2022). Overcoming these challenges necessitates interdisciplinary collaboration, technological advancement, ethical review, and strict regulatory compliance to enhance the feasibility and acceptance of the treatment, ultimately aiming to improve therapeutic outcomes for patients with thalassemia.

## 6 Conclusion

### 6.1 The current status and challenges of stem cell therapy

Currently, in the area of stem cell therapy, the management of thalassemia mainly relies on hematopoietic stem cells, whereas MSCs and iPSCs demonstrate considerable promise for a range of uses (Muthu et al., 2022). Nonetheless, there are variations in both therapeutic effectiveness and safety among stem cells derived from different origins. For example, MSCs obtained from umbilical cord blood present advantages like reduced immunogenicity (Um et al., 2020); however, they may not exhibit the same level of cell activity and functionality as MSCs obtained from bone marrow (Shang et al., 2021). Despite their low immunogenicity, MSCs can still trigger immune responses under certain conditions, and the performance and longevity of stem cells *in vivo* can be affected by factors such as inflammation and hypoxia (Chen et al., 2023). Furthermore, the attributes and size of the patient cohort (including factors like age, severity of the disease, and genetic background) might also influence the comparability of the research findings (Česnik et al., 2024). The constraints of existing technologies, such as the accuracy of gene-editing instruments and inconsistencies in cell culture conditions, can also contribute to varying results (Česnik and Švajger, 2024; Mushahary et al., 2018). Although advancements have been achieved, obstacles continue to exist: iPSCs encounter problems related to epigenetic instability and the potential for tumor formation, whereas adult stem cells struggle to accomplish successful differentiation (Su et al., 2025). Ongoing assessment of long-term safety and effectiveness is essential for further verification.

### 6.2 The capabilities and constraints of gene-editing technology

Regarding gene-editing technology, CRISPR/Cas9 has demonstrated remarkable promise in correcting gene mutations related to thalassemia, but there are still significant differences in off-target effects and editing efficiency across different studies. Some report high editing efficiency but note potential off-target effects, whereas others manage to minimize off-target effects but still face challenges in enhancing editing efficiency (Zeng et al., 2023). These inconsistent outcomes may result from differences in experimental design, cell sources, gene-editing methods, or animal models, given that viral vectors have high delivery efficiency but are associated with risks such as immune reactions and insertional mutations, whereas nonviral delivery systems, although safer, tend to have lower efficiency (Taghdiri and Mussolino, 2024). The potential impact of the limitations of current technologies on research outcomes cannot be ignored. Despite advancements in CRISPR/Cas9 technology aimed at minimizing off-target effects, including the development of high-fidelity Cas9 variants and enhanced sgRNA designs, the total eradication of off-target effects remains a significant challenge (Guo et al., 2023).

### 6.3 Future directions for the development of thalassemia treatment technologies

To address these challenges, future studies should implement more standardized experimental frameworks and methods to minimize variability in outcomes while simultaneously promoting the advancement of safer and more effective gene-editing technologies and delivery systems (Lino et al., 2018). Furthermore, the significance of collaboration across multiple disciplines should not be underestimated, as integrating knowledge from gene editing, cell biology, clinical practice, and other areas can collaboratively enhance the progression of thalassemia treatment technologies (Christakopoulos et al., 2023).
